# Symbols Are Special: An fMRI Adaptation Study of Symbolic, Nonsymbolic, and Non-Numerical Magnitude Processing in the Human Brain

**DOI:** 10.1093/texcom/tgab048

**Published:** 2021-07-23

**Authors:** H Moriah Sokolowski, Zachary Hawes, Lien Peters, Daniel Ansari

**Affiliations:** Rotman Research Institute, Baycrest Hospital, North York, ON M6A 2E1, Canada; Ontario Institute for Studies in Education, University of Toronto, Toronto, ON M5S1V6, Canada; Numerical Cognition Laboratory, Department of Psychology & Brain and Mind Institute, University of Western Ontario, London, ON N6A 3K7, Canada; Numerical Cognition Laboratory, Department of Psychology & Brain and Mind Institute, University of Western Ontario, London, ON N6A 3K7, Canada

**Keywords:** fMRI, human uniqueness, magnitude processing, neural adaptation, nonsymbolic, numerical cognition, symbolic

## Abstract

How are different formats of magnitudes represented in the human brain? We used functional magnetic resonance imaging adaptation to isolate representations of symbols, quantities, and physical size in 45 adults. Results indicate that the neural correlates supporting the passive processing of number symbols are largely dissociable from those supporting quantities and physical size, anatomically and representationally. Anatomically, passive processing of quantities and size correlate with activation in the right intraparietal sulcus, whereas symbolic number processing, compared with quantity processing, correlates with activation in the left inferior parietal lobule. Representationally, neural patterns of activation supporting symbols are dissimilar from neural activation patterns supporting quantity and size in the bilateral parietal lobes. These findings challenge the longstanding notion that the culturally acquired ability to conceptualize symbolic numbers is represented using entirely the same brain systems that support the evolutionarily ancient system used to process quantities. Moreover, these data reveal that regions that support numerical magnitude processing are also important for the processing of non-numerical magnitudes. This discovery compels future investigations of the neural consequences of acquiring knowledge of symbolic numbers.

## Introduction

As a species, humans are unique in our ability to represent numerical magnitudes symbolically (e.g., “3” or “three”). The exceptional capacity to understand and manipulate numerical symbols is necessary for mathematical thinking; a pillar of contemporary civilization. The ability to understand symbolic numerical magnitudes is thought to be supported by the same brain regions that are associated with a preexisting, innate, and evolutionarily ancient abstract numerical magnitude processing system used to process nonsymbolic numerical magnitudes, often referred to as quantities (e.g., three dots “•••”) (Pansky and Algom 2002; Dehaene et al. 2003; Brannon 2006; Dehaene 2007; Nieder and Dehaene 2009; Szkudlarek and Brannon 2017; Castaldi et al. 2019). However, a growing body of recent evidence suggests that brain regions used to process symbolic and nonsymbolic numerical magnitudes are more distinct than has been previously assumed (Ansari 2007; Cohen Kadosh and Walsh 2009; Lyons et al. 2012; Lyons and Beilock 2013; Bulthé et al. 2014; Lyons et al. 2014; Sokolowski and Ansari 2016), thus conflicting with the notion that numbers are processed entirely abstractly. Despite years of research, and a recent meta-analysis of neuroimaging papers (Sokolowski, Fias, Mousa, et al. 2017), the degree of the dissociation in the way the brain processes symbolic compared with nonsymbolic numerical magnitudes remains unknown (Cohen Kadosh and Walsh 2009; Piazza and Izard 2009; Piazza and Eger 2016; Wilkey and Ansari 2019).

Brain regions associated with numerical magnitude processing are also activated during the processing of non-numerical magnitudes such as physical size, duration, and luminance (Walsh 2003; Cohen Kadosh et al. 2008; Cantlon et al. 2009b; Sokolowski, Fias, Ononye, et al. 2017). This finding of common brain regions supporting numerical and non-numerical magnitude processing has been taken to suggest that the common brain regions used to process both symbolic and nonsymbolic numerical magnitudes (often referred to as an abstract number processing system) may be a general system used to process both numerical and non-numerical magnitudes. Few neuroimaging studies examining nonsymbolic stimuli sufficiently control for continuous properties of the nonsymbolic stimuli (e.g., controlling for the area of space taken up by objects with different quantities). Therefore, the question of whether symbolic and nonsymbolic numerical magnitudes are processed using the same brain regions while controlling for brain regions associated with non-numerical magnitude processing must still be addressed.

An additional challenge when addressing the question of whether distinct formats of numerical magnitudes are supported by overlapping neural systems is that the vast majority of studies that compared the neural correlates of symbolic and nonsymbolic numerical thinking used active tasks. When using active tasks, it is notoriously difficult to discern whether neural activation is associated with processing the magnitude of the stimulus or with decision-making and motor processing required to complete the active task (Göbel et al. 2004). Additionally, it is challenging to equate difficulty levels on active tasks, which means that a comparison of task effects of active tasks may reflect relative levels of difficulty rather than representational differences between the tasks. To overcome these limitations of active tasks, a small subset of research has used functional magnetic resonance imaging adaptation (fMR-A). fMR-A is a passive design that measures the neural correlates associated with stimuli of interest without requiring participants to make a decision or motor response. fMR-A relies on the principle that neural populations habituate (i.e., adapt) their activity following repeated presentations of the same stimulus (Grill-Spector et al. 2006). In fMR-A paradigms, a particular stimulus (i.e., the habituation stimulus) is repeatedly presented to evoke adaptation of brain regions associated with encoding this stimulus. Following this period of adaptation, a stimulus that differs in some way from the habituation stimulus (i.e., a deviant stimulus) is presented. The presentation of this deviant stimulus results in a rebound of activation in specific brain regions that are associated with the attributes of the particular deviant. In other words, the brain regions that support the aspect of the stimulus that differs between the habituation and deviant stimulus exhibit a neural rebound of activation in response to a deviant, referred to as the “neural rebound effect.” The size of the neural rebound effect in response to a deviant is a function of the difference between the adapted stimulus and the deviant. Within the number domain, the neural rebound effect is dependent on the “numerical distance” between the habituation and deviant stimulus (e.g., Vogel et al. 2017). For example, if a participant is adapted to the symbolic number “6” the neural rebound effect associated specifically with magnitude processing will be greater for a symbolic number deviant stimulus that is farther from the adapted stimulus (e.g., “9”) compared with a symbolic number that is closer to the adapted stimulus (e.g., “7”). Therefore, calculating a “neural distance effect” by subtracting activation in response to deviants with close numerical distances from activation in response to deviants with a far numerical distance allows us to identify regions specifically associated with magnitude processing (Pinel et al. 2004; Lyons and Ansari 2009; Holloway et al. 2010; Notebaert et al. 2010; Notebaert et al. 2011).

Using fMR-A, researchers have discovered that regions in the bilateral intraparietal sulcus (IPS) support symbolic and nonsymbolic numerical magnitude processing (Piazza et al. 2004; Cohen Kadosh et al. 2007; Piazza et al. 2007; Roggeman et al. 2007; Notebaert et al. 2011; Damarla and Just 2013; Holloway et al. 2013; Demeyere et al. 2014; Vogel et al. 2017). A meta-analytic synthesis that included many of these passive fMR-A tasks revealed convergent activation for the passive processing of numerical symbols in the left inferior parietal lobule and convergent activation for the passive processing of nonsymbolic numerical magnitudes in bilateral regions of the parietal lobes (Sokolowski, Fias, Mousa, et al. 2017). This indicates that both overlapping and distinct brain regions support the processing of symbolic and nonsymbolic numerical magnitudes in the absence of task demands. However, the majority of the studies included in the passive viewing meta-analysis include only a symbolic (Cohen Kadosh et al. 2007; Notebaert et al. 2011; Price and Ansari 2011; Holloway et al. 2013; Vogel et al. 2017) or a nonsymbolic condition (Piazza et al. 2004; Ansari et al. 2006; Cantlon et al. 2006; Jacob and Nieder 2009; Roggeman et al. 2011; Demeyere et al. 2014) but not both conditions. Without the inclusion of both a symbolic and nonsymbolic condition within a single controlled sample of participants, it is challenging to determine the degree to which the systems supporting symbolic and nonsymbolic number processing are overlapping. There are a few key studies that have developed innovative fMR-A paradigms that include both symbols and quantities and use these paradigms to examine the passive processing of both symbolic and nonsymbolic numerical magnitudes using fMR-A (e.g., Piazza et al. 2007; Roggeman et al. 2007; Cohen Kadosh et al. 2011; Damarla and Just 2013). The adaptation paradigms in these studies involved habituating participants to either symbolic or nonsymbolic numbers and then presenting deviants in either the same format (e.g., habituate to a symbolic numerical magnitude then present a symbolic deviant) or distinct format (e.g., habituate to a symbolic numerical magnitude then present a nonsymbolic deviant). This cross-format adaptation design allowed researchers to make inferences about whether semantic numerical representations of one format are generalizable to another. As with the studies that used active tasks, some studies suggest that numerical representation is subserved by entirely overlapping brain regions, suggested to reflect a single abstract number processing mechanism, instantiated in the bilateral parietal lobes (e.g., Piazza et al. 2007), whereas others indicate distinct brain regions support symbolic and nonsymbolic numerical magnitudes (e.g., Cohen Kadosh et al. 2011).

We address this fundamental question of whether the culturally acquired, uniquely human ability to process numbers symbolically is underpinned by the same brain regions that are activated during the processing of nonsymbolic quantities and physical size using a novel adaptation paradigm, inspired by cross-format adaptation. In the present preregistered study (https://osf.io/jrmpf/register/5771ca429ad5a1020de2872e), we develop and implement “parallel fMR-A” to isolate and directly compare the neural representations of symbols, quantities, and physical size. Specifically, in our parallel fMR-A design, participants are repeatedly presented with a specific quantity of the same symbolic number in a white-colored font of a specific size. This set of symbols will be referred to as an “array.” Following this, one aspect of the array is changed (either the symbol, the quantity, or the size), whereas the other aspects remain constant. As with other adaptation tasks, parallel adaptation overcomes inherent confounds associated with active tasks (Grill-Spector et al. 2006). However, our design adds several important additional controls. In cross-format adaptation designs, assessing magnitude change across formats requires that the magnitude and format deviate simultaneously. By adapting participants to multiple formats in parallel, the parallel fMR-A paradigm disconfounds format from magnitude, allowing us to measure brain regions associated with format-specific processing of magnitude rather than magnitude processing across formats. Additionally, the inclusion of a physical size condition in the parallel fMR-A task allows us to identify whether the brain regions that support numerical magnitude processing are number specific or associated with magnitude more generally. Finally, rather than using a region of interest (ROI) approach, the current study canvasses the whole brain in search of brain regions that support the passive processing of numerical and non-numerical magnitudes. In summary, this design allows us to identify overlapping and distinct brain regions associated with the passive processing of symbolic, nonsymbolic, and non-numerical magnitudes, in the adult brain.

## Methods

### Participants

Fifty-two healthy adult participants from London, Ontario, Canada participated in the fMR-A experiment. Our final sample included 45 participants (mean_age_ = 23.6, standard deviation [SD]_age_ = 4.3, age range = 18–39; 30 women and 22 men), all of whom did not exceed our motion cutoffs (i.e., no overall deviation >3 mm from the first volume acquired within a run, and no deviation >1.5 mm between subsequent volumes) and our accuracy cutoffs (Vogel et al. 2015). Accuracy was determined by asking participants to press a predefined button with their right index finger when the numbers appeared in blue font. These trials are referred to as “catch trials.” The runs where the participant did not “catch” at least 5 out of 7 trials were excluded from analyses. Participants with <2 out of 3 usable runs were excluded from the study. All included participants were right-handed, spoke fluent English, reported no known history of psychiatric or neurological disorders, and had normal or corrected to normal vision. The 7 excluded participants were excluded due to exceeding preregistered motion cutoffs (*n* = 4), being left-handed (*n* = 1), misunderstanding instructions and pressing the button on all trials rather than catch trials only (*n* = 1), and an incidental finding that prohibited coregistration (*n* = 1). The procedures of this study were approved by the Health Sciences Research Ethics Board for human subjects at the University of Western Ontario. Experiments were undertaken with the understanding and written consent of each subject.

### Stimuli

**Figure 1:**
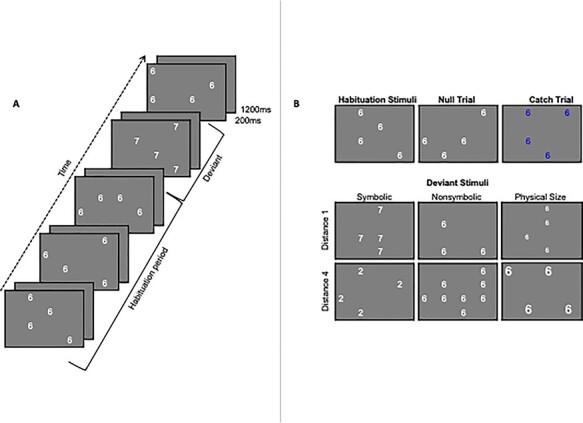
(*A*) Example of the parallel adaptation paradigm: including the continuous presentation of the adapted stimulus (habituation period) followed by a deviant stimulus (in this case a symbolic deviant). (*B*) Illustrations of examples of the adaptation stimulus, six deviant stimuli types (symbolic distance 1, symbolic distance 4, nonsymbolic distance 1, nonsymbolic distance 4, physical size small change, and physical size large change), and catch trial types (i.e., trials for which participants were instructed to press a button, to assure a minimum degree of attentiveness toward the stimuli presentation in the scanner).

Stimuli were created using MATLAB (Fig. 1*A*). The code to create the stimuli is available on the open science framework (OSF) at https://osf.io/9gfj4/. Habituation stimuli contained white “6”s in font size 60 on a gray background (see Fig. 1*B* for an example of a habituation array). Participants were simultaneously adapted to 3 aspects of the array: the numerical symbol (i.e., “6”), the quantity (i.e., the quantity of “6”s displayed), and the physical size of the digits. Deviant stimuli (i.e., stimuli that differed from the habituation stimuli in a particular way) were variations of an array of white Arabic digits randomly positioned on a gray background (Fig. 1*B*). Catch trials (i.e., trials for which participants were instructed to press a button) contained Arabic digits printed in blue on the same gray background. As previously stated, to meet our accuracy cutoffs, participants were required to “catch” at least 5 out of the 7 trials per run (Vogel et al. 2015). Multiple versions of the array for each condition were generated to ensure that participants did not learn the position of the Arabic digits within the array. E-prime 2.0 presentation software (Schneider et al. 2002) was used to project the stimuli onto a computer screen (resolution = 800 × 600 pixels; color bit depth = 16). The paradigm is available at https://osf.io/gx63r/. The participants viewed the computer screen using a mirror system that was attached to the magnetic resonance imaging (MRI) head-coil.

### Experimental Procedure

The fMR-A task was modeled after previous adaptation studies (Holloway et al. 2013; Vogel et al. 2015, 2017). Participants were instructed to attend to the screen and press a button when the digits on the screen turned blue (i.e., catch trials). The experiment included 3 fMR-A runs, each consisting of a stream of arrays of Arabic digits in Helvetica font punctuated by blank gray screens that were the same color as the background of the arrays. The arrays were presented for 200 ms and the blank gray screen for 1200 ms (Fig. 1*A*). During habituation, participants were presented with the digit “6” in 4 random locations of the screen in size 60 font between 5 and 9 times (average of 7 repeats). This allowed for a natural oversampling of the hemodynamic response function as the presentation of 1 trial (1400 ms) was not synchronized with the scan repetition time (TR = 1000 ms). At jittered intervals (i.e., after 5–9 habituation trials), participants were presented with either a deviant trial (48 total trials across 6 conditions), a null trial (9 total), or a catch trial (7 total). In deviant trials, one aspect of the array of sixes was changed a small amount or a large amount. There were 6 conditions of deviant trial types (8 trials per deviant). Specifically, there were 3 types of deviants (symbolic, nonsymbolic, physical size), and each type changed a large amount or a small amount (small change, large change). In the symbolic condition, the numerical symbols changed from “6”s to “7”s (small change) or to “2”s (large change), whereas the quantity and physical size were held constant. In the nonsymbolic condition, the quantity changed from four to three (small change) or 8 (large change) “6”s, but the symbol and physical size were held constant. For symbolic and nonsymbolic deviant conditions, the small change was a distance of 1 and the large change was a distance of 4. In the physical size condition, the size of the symbols decreased to font size 51 (small change) or increased to font size 86 (large change), but the symbol and quantity (i.e., four “6”s) remained unchanged. Critically, for the physical size condition, the area of the four digits was matched to the area taken up by the three digits in the quantity small change condition or the 8 digits in the quantity large change condition. Specifically, the number of white pixels in the physical size condition was matched to the corresponding nonsymbolic deviant conditions using MATLAB. The code is available at https://osf.io/rncv7/. In null trials, the participant was presented with another habituation trial array (i.e., four “6”s in size 60 font). These null trials were modeled separately from the adaptation trials and used as a “deviant” stimulus for which we predicted that there should be no neural rebound effect. In the catch trials, participants were presented with one of the six deviant trials or a null trial in blue font. Participants pressed a button with the index finger of their right hand when the digits on the screen turned blue (i.e., catch trials). Catch trials were pseudorandomly dispersed throughout each run and were not included in the modeling of the hemodynamic response function. Participants had to push the button for at least five of the seven catch trials for the run to be included in the statistical analyses. See Figure 1*B* for an illustration of the adaptation, deviant, null, and catch trials.

### Method of Selection of Numerical Stimuli

Due to the trade-off between the variety of stimuli and attentional time constraints, it was necessary to select a representative subset of stimuli that can be used to address our key questions. Numerical magnitudes included in the current study needed to be able to be represented with a single digit (i.e., 1–9), as this experiment involved the presentation of an array of digits as a condition of interest (nonsymbolic condition). Additionally, numerical magnitudes 1 and 9 were avoided as quantities at the edge of a set have been reported to behave differently than other numerical stimuli (e.g., Goldfarb et al. 2011).

#### Habituation Stimuli

The stimuli four and six were chosen to be the habituation stimuli. These numerical magnitudes are near the middle of the range of possible single-digit numerical magnitudes (1–9), allowing for both a large and small numerical distance between the habituation and deviant stimuli, and so deviants can be greater or smaller than the habituation stimulus. This habituation stimulus also ensured that the numerical magnitudes in the habituation condition were not the same across formats (e.g., displaying five of the digit “5”). This is necessary because participants respond differently to congruent (i.e., symbolic and nonsymbolic are the same magnitude) and incongruent (i.e., symbolic and nonsymbolic have different magnitudes) arrays of symbols (Pavese and Umiltà 1998; Pavese and Umiltà 1999; Furman and Rubinsten 2012). This means that any automatic inhibition that occurs due to the stimuli having two numerical dimensions that are incongruent is consistent between the habituation and deviant stimuli. Thus, the habituation condition acts as a control for potential congruity effects within the stimuli.

#### Deviant Stimuli

Deviant stimuli included a deviant with a numerical distance that was close (i.e., distance 1) and far (i.e., distance 4) for each condition. Specifically, in the symbolic small change condition, the four sixes changed to four sevens; in the symbolic large change condition, the four sixes changed to four twos; in the nonsymbolic small change condition, the four sixes changed to three sixes; and in the nonsymbolic large change condition, four sixes changed to eight sixes. In the symbolic condition, the small and large conditions differed in whether they were increasing or decreasing as compared with the nonsymbolic and physical size conditions. Tuning curves from previous empirical adaptation studies consistently reveal that the neural rebound in response to deviant stimuli is greater for deviants that are numerically more distant from the adapted stimulus, for both symbolic and nonsymbolic numerical magnitudes, regardless of if the deviant is increasing or decreasing (e.g., Piazza et al. 2004; Cohen Kadosh et al. 2007; Jacob and Nieder 2009; Holloway et al. 2013). Based on this consistent finding that increasing and decreasing sides of tuning curves are symmetrical, there is no strong prior that this should influence or confound our analyses of interest in any meaningful way. However, we include several control analyses to explicitly assess whether neural rebound effects are influenced by the direction of the deviant within these data.

### fMRI Data Acquisition

Structural and functional images were acquired using a 3 T Siemens Prisma Fit whole-body MRI scanner, using a 32-channel receive-only head-coil (Siemens, Erlangen Germany). A whole-brain high-resolution *T*1-weighted anatomical scan was collected using an MPRAGE sequence with 192 slices and a scan duration of 5 min and 21 s (isovoxel resolution = $1\times 1\times 1$; TR = 2300 ms; TE 2.98 = ms; TI = 900 ms; FOV = 256 mm; flip angle = 9°). Functional MRI data were acquired using a blood oxygen level–dependent (BOLD) sensitive *T*2* echo-planar (EPI) sequence. The *T*2-weighted functional scan was collected using a bold sequence with 48 slices and a scan duration of 12 min and 58 s. The 48 slices were acquired in a sequential multi-slice interleaved series with a multi-band accelerator factor of 4 (FIX voxel size = 2.5 × 2.5 × 2.5 mm; slice thickness = 2.5 mm; TR = 1000 ms; TE 30.00 = ms; FOV = 208 mm; flip angle = 40°). The scan has a base resolution of 84, a phase resolution of 100%, and a phase partial Fourier of 7/8. All defaced neuroimaging data are publicly available at https://openneuro.org/datasets/ds001848/versions/1.0.1.

### fMRI Data Preprocessing

Structural and functional data were preprocessed and analyzed in Brain Voyager 20.6 (Brain Innovation, Maastricht, the Netherlands) using the software’s preprocessing workflow (for workflow see: https://osf.io/3hr2g/). The structural brain data were extracted from the head tissue, and intensity inhomogeneities were corrected to reduce the spatial intensity of the 3D volumes. Functional data were corrected for slice-scan time acquisition (cubic-spline interpolation algorithm), high-pass filtered (Fourier; cutoff value of 2 sines/cosines cycles), in which a Fourier basis set is used to filter the design matrix and corrected for in-scanner head motion (Trilinear/sinc interpolation). A Gaussian smoothing kernel of 6-mm full-width at half-maximum (FWHM) was applied to smooth the images. Structural and functional images were coregistered using a header-based initial alignment followed by a gradient-driven fine-tuning adjustment and normalized to MNI-152 space. A 2-gamma hemodynamic response function was used to model the expected bold signal (Friston et al. 1998). Baseline was calculated using the adaptation period as well as the between trial fixation periods. Runs that had an overall deviation >3 mm from the first volume acquired within the run, or deviation >1.5 mm between subsequent volumes were removed from analyses, and therefore, motion parameters were not included as predictors of no interest. Catch trials were modeled as a predictor of no interest.

### Data Analysis

#### Statistical Threshold

All of the statistical maps reported in the current study were thresholded with an uncorrected *P* value of 0.001 (Woo and Wager 2014; Eklund et al. 2016). All statistical whole-brain maps were corrected for multiple comparisons at a statistical level of *P* < 0.05 using the cluster-level correction plugin in BrainVoyager (Forman et al. 1995). The FWHM in units of functional voxels (i.e., the smoothness), as well as the minimum cluster size (*P* = 0.05) based on the log-linear intra/extrapolation in millimeters (i.e., the cluster extent), are reported for each contrast with clusters of activation that reached a minimum threshold of *P* < 0.001, uncorrected and *P* < 0.05 cluster corrected at the whole-brain level (Woo and Wager 2014; Eklund et al. 2016).

#### Whole-Brain Analyses

Whole-brain random-effects analyses (RFX) were conducted using a general linear model to examine overlapping and distinct BOLD responses to symbolic numerical magnitudes, nonsymbolic numerical magnitudes, and the magnitude of physical size. Stimulus conditions within contrasts were weighted to ensure that the contrasts were balanced. For example, when comparing symbolic to both nonsymbolic and physical size the symbolic condition was weighted by a factor of two. All primary analyses were preregistered on the OSF (see https://osf.io/jrmpf/register/5771ca429ad5a1020de2872e for preregistration).

## Results

### Preregistered Analyses

The procedure and complete analysis plan for the current study were preregistered on the OSF (https://osf.io/jrmpf). The results presented follow this preregistered plan, in sequential order. Neural distance effects (i.e., neural rebound in response to a large distance deviant compared with a small distance deviant) are used to address the core research questions as they allow us to identify changes in magnitude independently from format changes.

#### Change Detection

Preliminary contrast analyses were conducted to identify brain regions that responded to changes in different stimulus dimensions. Regions that were associated with stimulus change detection were identified as regions associated with the change of one stimulus type (at both distances) over the change of the other two stimulus types (at both distances); for example, the symbolic change effect is calculated as [(symbolic distance 1 + symbolic distance 4) > (nonsymbolic distance 1 + nonsymbolic distance 4 + physical size distance 1 + physical size distance 4)]. The stimulus conditions were weighted to ensure that the contrast was balanced for all contrasts. Results from these change detection analyses cannot inform our primary research questions, as they do not identify regions associated with magnitude. Instead, these analyses were included as a preliminary assessment to examine if participants do exhibit a neural rebound effect after adapting to multiple aspects of an array in parallel.

Results revealed that symbolic change detection (cluster-level: smoothing = 2.49; extent = 462 mm) associated with activation in a widespread frontal–parietal–occipital network (Table 1). There were no brain regions that were activated above the threshold in response to nonsymbolic change detection. Physical size change detection (cluster-level: smoothing = 2.25; extent = 373 mm) associated with activation in the right inferior parietal lobule, and left visual cortex (Table 1). Although these preliminary analyses highlight regions that correlate with the passive perception of change detection within each format, these brain regions are not specifically associated with the magnitude processing of symbols, quantities, and physical size. The critical analyses to address how the human brain processes the magnitude in different formats require assessing neural rebound effect at distance 4–distance 1 (i.e., distance effects).

**Table 1:** Brain regions associated with change detection signal recovery from adaptation

Hemi-sphere	Brain region		Peak MNI coordinate			*t*	*P*	Cluster size
	Juelich histological atlas	Harvard–Oxford structural atlas	*x*	*y*	*z*			(Number of voxels)
		*Symbolic change detection*						
R	Anterior intraparietal sulcus	Superior parietal lobule, angular gyrus	33	−52	43	5.46	0.000002	5242
R	Callosal body, cingulum	Cingulate gyrus	3	−34	28	4.46	0.00006	755
L		Cerebellum	−6	−89	−32	4.45	0.00006	534
L	Anterior intraparietal sulcus, superior parietal lobule	Lateral occipital cortex, superior parietal lobule, angular gyrus	−30	−61	46	4.44	0.00006	836
L		Temporal occipital fusiform cortex, inferior temporal gyrus	−36	−55	−23	4.58	0.0004	643
L	Visual cortex V4	Lateral occipital cortex, occipital fusiform gyrus	−45	−76	−17	5.80	0.000001	891
		*Nonsymbolic change detection*						
—	—	—	—	—	—	—	—	—
		*Physical size change detection*						
R	Inferior parietal lobule	Supramarginal gyrus	60	−37	22	4.46	0.00006	432
L	Visual cortex	Lateral occipital cortex, occipital pole	−30	−88	−2	4.51	0.00005	591

#### Neural Distance Effects

**Figure 2:**
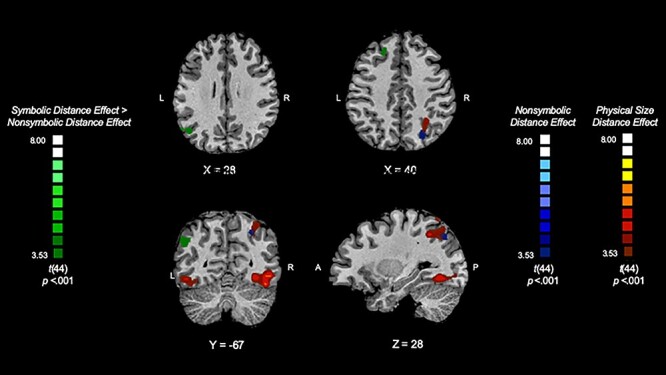
The neural rebound effects for nonsymbolic numerical magnitude processing (nonsymbolic distance 4 deviant > nonsymbolic distance 1 deviant), shown in blue, physical size magnitude processing (physical size large change deviant > physical size small change deviant), shown in red, and symbolic numerical magnitude processing > nonsymbolic numerical magnitude processing [(symbolic distance 4 deviant > symbolic distance 1 deviant) > (nonsymbolic distance 4 deviant > nonsymbolic distance 1 deviant)], shown in green. This reveals the brain regions associated with the processing of nonsymbolic and physical size magnitude and a region that reflects spatial separation for symbolic numerical magnitude processing.

Neural distance effects allow us to compare the brain regions that repond to magnitude in the three different formats. We examined neural distance effects (i.e., distance 4 > distance 1) to isolate brain regions associated with magnitude processing, of each deviant stimulus type (symbolic, nonsymbolic, physical size). Specifically, we statistically compared distance 4 to distance 1 for the symbolic condition (symbolic distance 4 > symbolic distance 1), the nonsymbolic condition (nonsymbolic distance 4 > nonsymbolic distance 1), and the physical size condition (physical size large change > physical size small change). Symbolic magnitude processing was associated with small clusters of activation in the left inferior parietal lobule (peak MNI coordinate: −57, −64, 22) and the left frontal orbital cortex (peak MNI coordinate: −36, 35, −14), but these clusters did not survive cluster correction. Distinct from this, nonsymbolic magnitude processing (cluster-level: smoothing = 2.26; extent = 375 mm) was associated with activation spanning the right intraparietal sulcus and superior parietal lobule (peak MNI coordinate: 27, −67, 40; *t* = 4.05, *P* = 0.0001; cluster size = 417 voxels) (Fig. 2). Physical size magnitude processing (cluster-level: smoothing = 2.45; extent = 442 mm) associated with activation spanning the right intraparietal sulcus and inferior parietal lobe (peak MNI coordinate: 33, −52, 43; *t* = 5.49, *P* = 0.000002; cluster size = 2718 voxels) and regions in the right occipital fusiform gyrus (peak MNI coordinate: 42, −61, −11; *t* = 5.87, *P* = 0.000001; cluster size = 9191 voxels) and left lateral occipital cortex (peak MNI coordinate: −45, −67, −11; t = 4.79, *P* = 0.00002; cluster size = 1076 voxels) (Fig. 2).

Reverse distance effects (i.e., distance 1 > distance 4) for each condition were also examined as control analyses to ensure that the distance-dependent activation correlated with greater magnitude. Results revealed that no brain regions were more activated in response to distance 1 compared with distance 4 for the symbolic and physical size conditions and 1 brain region associated with nonsymbolic distance 1 > nonsymbolic distance 4 in the visual cortex (peak MNI coordinate: 0, −79, 22; *t* = −4.18, *P* = 0.0001; cluster size = 466 voxels)**.**

Following our preregistered analysis plan, we next used a conjunction (∩) of RFX to assess whether the brain regions associated with symbolic, nonsymbolic, and physical size magnitude processing overlapped. This analysis [(symbolic distance 4 > symbolic distance 1) ∩ (nonsymbolic distance 4 > nonsymbolic distance 1) ∩ (physical size large change > physical size small change)] revealed that there are no brain regions commonly activated by symbolic, nonsymbolic, and physical size magnitude processing.

To identify which brain regions support numerical magnitude processing specifically, the conjunction of the symbolic and nonsymbolic distance effects were contrasted against the physical size distance effect [(symbolic distance 4 ≥ symbolic distance 1) ∩ (nonsymbolic distance 4 > nonsymbolic distance 1) > (physical size large change > physical size small change)]. No brain regions were significantly activated for numerical magnitude processing (symbolic and nonsymbolic) over and above brain regions associated with physical size processing.

For the final set of preplanned analyses we examined the conjunction of each distance effect compared with the distance effects of the other conditions to identify specific brain regions associated with the processing of symbolic, nonsymbolic, and physical size magnitudes; for example, for symbolic specific magnitude processing: [(symbolic distance 4 > symbolic distance 1) > (nonsymbolic distance 4 > nonsymbolic distance 1) ∩ (symbolic distance 4 > symbolic distance 1) > (physical size large change > physical size small change)]. These analyses resulted in no significant brain regions.

### Exploratory Analyses

Findings from preregistered analyses hint that symbols might be processed partially distinctly from quantities and physical size in the parietal lobes. It is notable that preregistered analyses that addressed key predictions of overlap were all conjunction analyses that included all three conditions. More specifically, for any region to be considered distinct, our preregistered analyses required that condition to be more strongly activated in a given brain region than the other two conditions. Similarly, for two regions to be considered overlapping, the overlap had to be significant over and above activation of the third condition.

Given that our preregistered analysis revealed substantial overlap between physical size and nonsymbolic numerical magnitude processing, we wanted to investigate the similarities and difference between symbolic and nonsymbolic numerical magnitude processing in the absence of the physical size condition. Additionally, these preregistered findings highlight the need to explore the similarities and differences between nonsymbolic numerical magnitude processing and physical size in the absence of the symbolic number processing. Therefore, we include follow-up contrast analyses to further probe overlapping and distinct brain regions associated with 1) symbolic and nonsymbolic distance effects and 2) nonsymbolic and physical size distance effects.

#### Numerical Magnitude Processing

Exploratory contrast analyses were computed to directly compare brain modulation by distance for symbolic compared with nonsymbolic numerical magnitudes; that is, [(symbolic distance 4 > symbolic distance 1) > (nonsymbolic distance 4 > nonsymbolic distance 1)] and its symmetrical cluster-level: smoothing = 2.28; extent = 377 mm. Results revealed two brain regions that exhibited greater activation for the symbolic distance effect compared with the nonsymbolic distance effect, namely the left superior frontal gyrus (peak MNI coordinate: −21, 32, 43; *t* = 4.13, *P* = 0.0002; cluster size = 499 voxels) and left inferior parietal lobule, including the angular gyrus (peak MNI coordinate: −48, −64, 28; t = 4.45, *P* = 0.00006; cluster size = 908 voxels) (Fig. 2). There were no brain regions that exhibited greater activation for the nonsymbolic compared with the symbolic distance effect. An exploratory conjunction analysis used to compute overlap between the symbolic and nonsymbolic distance effects [(symbolic distance 4 > symbolic distance 1) ∩ (nonsymbolic distance 4 > nonsymbolic distance 1)] resulted in no brain regions of significant overlap.

#### General Magnitude Processing

Exploratory conjunction and contrast analyses were also run to identify overlapping and distinct brain regions associated with nonsymbolic and physical size distance effects. The contrast analyses revealed no brain regions that were significantly associated with nonsymbolic distance effect > physical size distance effect or with physical size distance effect > nonsymbolic distance effect. The conjunction analysis examining overlapping activation between nonsymbolic magnitude processing and physical size magnitude processing [(nonsymbolic distance 4 > nonsymbolic distance 1) ∩ (physical size large change > physical size small change)] resulted in small clusters in the right intraparietal sulcus (peak MNI coordinate: 30, −67, 40; t = 3.71, *P* = 0.0007; cluster size = 61 voxels) and right inferior temporal gyrus (peak MNI coordinate: 48, −58, −14; *t* = 4.04, *p* = 0.0002; cluster size = 49 voxels), but these clusters did not survive cluster correction.

Together, the preplanned combined with post hoc univariate analyses suggest that nonsymbolic magnitudes and physical size are processed in the right intraparietal sulcus, whereas symbols are processed in the left inferior parietal lobule, when contrasted against nonsymbolic numerical magnitude processing. While these analyses suggest some spatial distinction between symbolic and nonsymbolic numerical magnitude processing, the symbolic distance effect alone was not significant, and the right IPS was not significant for nonsymbolic numerical magnitude processing over and above symbolic numerical magnitude processing. Therefore, while symbolic and nonsymbolic numerical magnitude processing seems to be somewhat lateralized in the parietal cortex both formats may still activate overlapping regions in the right IPS. Moreover, while nonsymbolic and physical size magnitudes appear to overlap in the right IPS the statistical conjunction of nonsymbolic and physical size processing did not survive cluster correction and was not significant above symbolic numerical magnitude processing. This suggests that while symbolic and nonsymbolic numerical magnitude processing seems to be lateralized in the parietal cortex, both formats may still activate overlapping regions.

Univariate analyses do not allow us to conclude that the underlying representations are unrelated. To address this outstanding issue, we used a multivariate approach to identify similarities and differences in the patterns of neural activity for symbolic numerical magnitude processing, nonsymbolic numerical magnitude processing, and the processing of physical size. More specifically, we used the multivariate method representational similarity analysis (RSA) to extract information about distributed patterns of representations within ROIs in the brain. This method is valuable in advancing our understanding of similarities and differences in the underlying representations of symbolic, nonsymbolic, and non-numerical magnitudes, rather than coarsely estimating spatial overlap.

#### Representational Similarity Analyses

**Figure 3:**
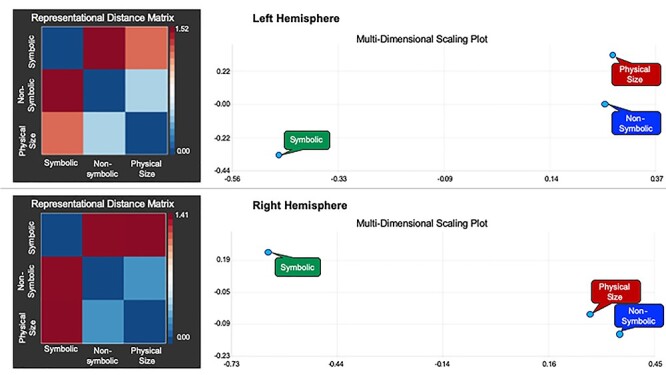
The left side of this figure illustrates the RDMs between the symbolic distance effect, the nonsymbolic distance effect, and the physical size distance effect in the left (top) and right (bottom) hemispheres. The numerical values that correspond to colors in the RDM refer to the distance measure calculated using the equation: *d* = 1 − *r*. Therefore, the values can range from 0.0 (minimum distance) to 2.0 (maximum distance) with the value “1.0” in the middle representing no correlation. The right side of this figure depicts the MDS plots, which are visualizations of the similarity between the 3 distance effects (symbolic, nonsymbolic, physical size) in 2D space. The MDS plot is a visualization of the distances between conditions in a 2D space that maximally satisfies the pairwise distances to all other conditions. The left and right parietal ROIs were derived from the weighted centre of the bilateral parietal clusters in the numerical passive viewing map (Sokolowski, Fias, Mousa, et al. 2017).

We implemented RSA using Brain Voyager 20.6 (Brain Innovation, Maastricht, the Netherlands) to analyze the similarity between evoked functional magnetic resonance imaging (fMRI) responses for the symbolic distance effect, the nonsymbolic distance effect, and the physical size distance effect in select ROIs. The ROIs were constructed by creating a sphere with a radius of 10 mm around the weighted centre of the bilateral parietal clusters in the numerical passive viewing map from Sokolowski, Fias, Mousa, et al. (2017). The coordinates for the weighted centre of the parietal clusters are 1) right hemisphere: MNI coordinates (*x*, *y*, *z*): 26, −55, 53; 2) left hemisphere: MNI coordinates (*x*, *y*, *z*): −28, −67, 43. For each ROI, a representational distance (or dissimilarity) matrix (RDM) was computed to assess the dissimilarity between the symbolic distance effect, the nonsymbolic distance effect, and the physical size distance effect (Fig. 3). Note that the correlation calculated between patterns reflects the similarity of the spatial patterns since this measure abstracts from the mean (and SD) of the original values. The RDM contains a cell for each pair of experimental conditions. The color of each cell represents a number that reflects the dissimilarities between the activity patterns associated with the two experimental conditions. Specifically, a Pearson correlation coefficient was calculated and subsequently transformed to a distance measure using the equation: *d* = 1 − *r*. These calculated *d* values, thus, range from 0.0 (minimum distance) to 2.0 (maximum distance) with the value “1.0” in the middle representing no correlation. These data are further visualized using multi-dimensional scaling (MDS) plots, which depict the similarity between the conditions in a 2D representation (Fig. 3). Specifically, the conditions that are positioned closer together on the MDS plot have more similar neural activation patterns. Notably, results from this multivariate analysis revealed that nonsymbolic magnitude processing and physical size processing correlate more strongly at the multivariate level than either do with symbolic magnitude processing in both the right and the left hemispheres. Notably, this pattern of greater similarity between nonsymbolic and physical size compared with symbols is especially strong in the right hemisphere. In sum, these multivariate results revealed a dissimilar normalized pattern of activation for symbolic compared with nonsymbolic numerical magnitude processing in both the left and right parietal lobes. Together the converging evidence from the univariate and multivariate analyses show that, in the adult human brain, symbols are processed using distinct brain regions, and distinct patterns of activation, compared with nonsymbolic and non-numerical magnitudes.

#### Assessing the Symmetry of Distance Effects

**Figure 4:**
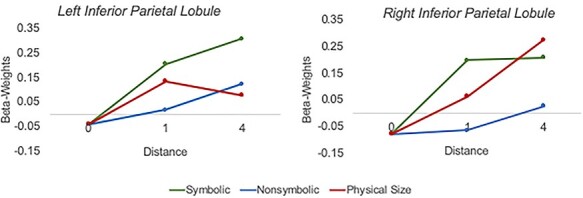
Plotted beta-weights for all deviants (null, symbolic distance 1, symbolic distance 4, nonsymbolic distance 1, nonsymbolic distance 4, physical size distance 1, physical size distance 4) in ROIs in the left inferior parietal lobule (left plot) and right inferior parietal lobule (right plot). ROIs were derived from the weighted centre of the bilateral parietal clusters in the numerical passive viewing map from Sokolowski, Fias, Mousa, et al. (2017).

The priority for the current study was to include a distance of 1 and 4 without having a double-digit deviant or a deviant of “zero” and avoiding the inclusion of any stimuli that were “congruent” (i.e., the symbolic and nonsymbolic stimuli being the same numerical magnitudes). This resulted in stimuli where the symbolic trials small change deviant is greater than the habituation stimulus, whereas the large change deviant is less than the habituation stimulus, whereas for nonsymbolic and non-numerical trials the paradigm is the reverse (large change is greater and small change is less than). This raises the legitimate question of whether or not the distance effects were symmetrical, despite the asymmetry of the directions of change. Analyses of reverse distance effects revealed no brain regions associated with magnitude processing responded to distance 1 > distance 4 for any condition. To further probe this potential confound, we plotted the beta-weights within the 10-mm spheres generated from the numerical passive viewing map from Sokolowski, Fias, Mousa, et al. (2017). These plots (Fig. 4) provide additional evidence that the distance 4–distance 1 contrast from the current paradigm does indeed reflect magnitude processing in all conditions.

## Discussion

The goal of the current study was to examine whether the uniquely human capacity to process symbolic numerical magnitudes relies on the same brain regions that support the processing of nonsymbolic numerical magnitudes (i.e., quantities) and/or non-numerical magnitudes (i.e., physical size). Parallel fMRI adaptation was used to isolate and directly compare the semantic representations of symbols, quantities, and physical size while controlling for neural activation associated with other conditions as well as for inherent confounds of active tasks (Grill-Spector et al. 2006). Key results revealed that nonsymbolic numerical magnitudes and non-numerical magnitudes (i.e., physical size) correlate with activation in the right intraparietal sulcus, whereas symbolic numerical magnitudes are specifically associated with a region in the left inferior parietal lobule, but only when contrasted against nonsymbolic numerical magnitudes. There were no brain regions that were significantly activated by magnitude processing of both symbols and quantities. The right IPS associated with nonsymbolic numerical magnitude processing but was not activated over and above symbolic numerical magnitude processing. The findings from the current study suggest that activation in the left parietal lobule is specific to symbolic number processing, whereas the right intraparietal sulcus is activated during nonsymbolic magnitude processing but also potentially to a lesser degree during symbolic numerical magnitude processing (Sokolowski, Fias, Mousa, et al. 2017). These findings align with previous research indicating that different number formats (symbolic and nonsymbolic) are lateralized within the parietal cortex (for review see: Sokolowski and Ansari 2016). At the multivariate level, normalized patterns of activation for symbolic numerical magnitude processing in both the left and right parietal lobes were distinct from patterns of activation for nonsymbolic magnitude processing. These findings align with conclusions from some previous studies that reveal qualitatively different coding of symbols compared with quantities in the brain (e.g., Bulthé et al. 2014; Lyons et al. 2014) but conflict with evidence revealing between-format generalization (e.g., Eger et al. 2009; Damarla and Just 2013; Wilkey et al. 2020). Therefore, the findings from the current study challenge the longstanding idea that symbolic numerical magnitudes are represented using entirely the same regions associated with the processing of nonsymbolic numerical magnitudes. Moreover, the findings from the current study suggest that a general magnitude system supports the processing of both discrete and continuous magnitudes, which is potentially also activated during the processing of symbols.

Broadly, findings from the current study contradict the longstanding, predominant view in the field that symbolic and nonsymbolic numerical magnitudes are supported by a single abstract number processing system (Dehaene et al. 1998; Brannon 2006; Piazza and Izard 2009; Cantlon et al. 2009a), and instead align with the view that symbolic and nonsymbolic numbers are processed using overlapping as well as distinct neural mechanisms (for review see: Cohen 2008; Sokolowski and Ansari 2016; Sokolowski, Fias, Mousa, et al. 2017). Moreover, the findings from the current study support this idea without typical confounds present in other studies. The parallel adaptation paradigm developed and employed in the present study overcomes major confounds of previous research that use active tasks such as decision-making and motor processing for these active tasks (Grill-Spector et al. 2006). Indeed, previously reported overlapping activation during the processing of symbolic and nonsymbolic numerical magnitudes could have resulted from overlapping task demands or the effortful process of mapping symbols onto quantities in the case of cross-format designs. Using our parallel adaptation approach, we discovered that the underlying brain systems supporting symbolic number processing differ from those that correlate with nonsymbolic magnitude processing in human adults, but not the reverse. Indeed, these data suggest that there is something special about symbols.

Results from the current study also show that the brain regions that are activated in response to nonsymbolic numerical magnitudes are highly similar to the regions that support the processing of non-numerical magnitudes, specifically physical size in the parietal lobe. Again, this finding that nonsymbolic numerical magnitudes and non-numerical magnitudes are supported by the same neural substrates directly contradicts the prevailing view in numerical cognition that symbolic and nonsymbolic numerical magnitudes are supported using an abstract number processing system that is specifically attuned to the processing of discrete quantities (Dehaene et al. 1998; Dehaene et al. 2003; Brannon 2006; Nieder and Dehaene 2009; Cantlon 2012). Instead, our findings indicate that the system used to process nonsymbolic numbers may be part of a general magnitude processing system used to process both discrete as well as continuous magnitudes (Walsh 2003; Cohen Kadosh et al. 2008; Lyons et al. 2012, 2014; Sokolowski, Fias, Ononye, et al. 2017).

Findings from the current study support the idea that symbolic number processing is supported by a partially dissociable system. Thus, we must consider the conceptual structure of a symbolic specific system. A key element that differentiates symbols from quantities is that without symbols, large quantities can only be represented approximately, whereas symbols can and in fact must be represented exactly. Therefore, while quantities may be processed using an analogue number system, in which the representations are noisy or approximate (Moyer and Landauer 1967; Dehaene et al. 1998; Dehaene 2007; Cantlon 2012) the processing of symbols may be supported by a different, more exact system. It has been suggested that symbols are understood based on their exact associations with other symbols (e.g., a symbol’s position in the counting row; “3” comes after “2” and before “4”) (for review see, Núñez 2017). The few behavioral and neuroimaging studies that have focused on uncovering the structure of the symbolic system propose that the system supporting the processing of symbolic numerical magnitudes may be best characterized by relational properties between symbols (Krajcsi et al. 2016; Lyons and Beilock 2018). Conceptually, this means that the symbolic number processing system might resemble a conceptual network or mental lexicon, rather than an analogue magnitude processing system. A related outstanding question that emerges from the current findings is “how did this unique symbolic system emerge across evolutionary and developmental time?” An alternate theory to the mapping of symbols onto an evolutionarily ancient abstract number processing system is that a general magnitude system (that evolved to compute continuous magnitudes [e.g., physical size]) was exploited for the emergence of the numerical system (Cantlon et al. 2009b; Henik et al. 2011; Leibovich et al. 2016). Based on evidence indicating that objects are organized within the visual cortex according to their real-world size (Konkle and Oliva 2012; Konkle and Caramazza 2013), it has been hypothesized that conceptual size might serve as a bridge between continuous magnitudes and discrete quantities, including those represented by symbols (Gabay et al. 2016). However, future research is needed to uncover the exact structure of a symbolic specific system across development and subsequently discover how this system emerges.

Here, we suggest the differences observed in the current study on the neural correlates supporting the processing of symbols and quantities are a consequence of these different formats being supported by distinct systems. However, it must be noted that within these systems these differences could be attributed to differences in representations, processing, or automaticity of the formats. While the current study cannot identify with certainty which of these potential explanations explains these findings, we believe that the results from the current study do highlight the need to consider these differences and develop novel paradigms that will enhance our understanding of what drives the difference in neural representations of symbols compared with quantities.

## Limitations

The current study has several limitations. First, as the stimuli consist of arrays that include both symbolic and nonsymbolic numerical magnitudes, the possibility that these different formats automatically influence each other during processing (e.g., Morton 1969; Pansky and Algom 2002; Naparstek and Henik 2010) cannot be ruled out. However, the fact that a neural distance effect was found for both symbolic and nonsymbolic deviants, in distinct brain regions for symbolic, suggests that the paradigm captured elements of magnitude processing that were specific to each format. Second, due to attentional time constraints of the participants, it was not possible to include multiple numerical values for the habituation stimulus and within deviant categories. Thus, the results from this study are specific to the particular magnitudes we included and should not be generalized to all numerical magnitudes. Notably, previous single-format adaptation studies that include a single stimulus type for the habituation stimulus that differ from the stimuli in the current study report highly similar adaptation effects (e.g., Notebaert et al. 2011; Holloway et al. 2013; Vogel et al. 2017). Relatedly, for the symbolic trials, small change deviant is greater than the habituation stimulus, whereas the large change deviant is less than the habituation stimulus, whereas for nonsymbolic and non-numerical trials the paradigm is the reverse (large change is greater and small change is less than). This decision was made to ensure that it was possible to include a distance of 1 and 4 without having a double-digit deviant or a deviant of “zero” and avoiding the inclusion of any stimuli that were “congruent” (i.e., the symbolic and nonsymbolic stimuli being the same numerical magnitudes). Given the many behavioral, neurophysiological, and neuroimaging studies suggesting symmetries in distance effects, ratio effects, and tuning curves (e.g., Piazza et al. 2004; Jacob and Nieder 2009; Holloway et al. 2013), there is no strong prior for this being a confounding variable. Moreover, control analyses of the current data reveal that activation in magnitude relevant regions increases as a function of numerical distance, regardless of whether the stimulus is increasing or decreasing. Future research is needed to examine whether the key findings from the current study that used parallel adaptation remain when including multiple different stimuli for the conditions for both habituation and deviant stimuli.

## Conclusions

This study provides evidence in support of the idea that the human adult brain processes symbolic numerical magnitudes using some brain regions that are quite distinct from those that support the processing of nonsymbolic numerical magnitudes. Specifically, symbols, as compared with quantities, are passively processed in the left parietal lobe, whereas quantities are processed the right parietal lobe, but not over and above symbols. The non-numerical magnitude, physical size, is also associated with brain activation in the right parietal lobe. RSA in the parietal lobes reveal that symbols are represented quite differently from quantities and physical size, which have similar patterns of activation to each other. These findings conflict with the dominant view in the field that symbolic and nonsymbolic numerical magnitudes are solely supported by a single abstract number processing system (Dehaene et al. 1998; Dehaene 2007; Nieder and Dehaene 2009; Cantlon 2012). Instead, data from the current study indicate that the human adult brain supports culturally acquired symbolic representations in a manner that is distinct from how the brain supports the evolutionarily ancient capacity to process nonsymbolic numerical magnitudes and the non-numerical magnitude, physical size. Our data highlight the need for the field of numerical cognition to shift away from conducting research with the goal of canvassing the brain in search of an abstract number processing system. Instead, efforts should be directed toward uncovering the multifaceted behavioral and neural consequences of learning the complex, uniquely human skill of symbolic abstraction.

## Notes

The researchers also acknowledge the Canada First Research Excellence Fund to BrainsCAN award. We would also like to thank the participants who volunteered their time to participate in this study. *Conflict of Interest*: None declared.
